# Current Advances in the Biodegradation and Bioconversion of Polyethylene Terephthalate

**DOI:** 10.3390/microorganisms10010039

**Published:** 2021-12-26

**Authors:** Xinhua Qi, Wenlong Yan, Zhibei Cao, Mingzhu Ding, Yingjin Yuan

**Affiliations:** 1 Frontier Science Center for Synthetic Biology and Key Laboratory of Systems Bioengineering (Ministry of Education), School of Chemical Engineering and Technology, Tianjin University, Tianjin 300072, China; xhqi@tju.edu.cn (X.Q.); wlyan@tju.edu.cn (W.Y.); caozhibei520@tju.edu.cn (Z.C.); yjyuan@tju.edu.cn (Y.Y.); 2 Collaborative Innovation Center of Chemical Science and Engineering (Tianjin), Tianjin University, Tianjin 300072, China

**Keywords:** polyethylene terephthalate, biodegradation, bioconversion, artificial microbial consortia

## Abstract

Polyethylene terephthalate (PET) is a widely used plastic that is polymerized by terephthalic acid (TPA) and ethylene glycol (EG). In recent years, PET biodegradation and bioconversion have become important in solving environmental plastic pollution. More and more PET hydrolases have been discovered and modified, which mainly act on and degrade the ester bond of PET. The monomers, TPA and EG, can be further utilized by microorganisms, entering the tricarboxylic acid cycle (TCA cycle) or being converted into high value chemicals, and finally realizing the biodegradation and bioconversion of PET. Based on synthetic biology and metabolic engineering strategies, this review summarizes the current advances in the modified PET hydrolases, engineered microbial chassis in degrading PET, bioconversion pathways of PET monomers, and artificial microbial consortia in PET biodegradation and bioconversion. Artificial microbial consortium provides novel ideas for the biodegradation and bioconversion of PET or other complex polymers. It is helpful to realize the one-step bioconversion of PET into high value chemicals.

## 1. Introduction

Polyethylene terephthalate (PET) is one of the most widely used synthetic plastics in people’s lives [1]. It is polymerized by terephthalic acid (TPA) and ethylene glycol (EG) through ester bonds [2]. Since PET was first used to produce disposable plastic bottles in the 20th century, it has been welcomed worldwide and has become an indispensable part of people’s lives [3]. As PET is highly resistant to natural degradation, the recycling of PET has been encouraged [4]. At present, the main methods for managing PET waste include landfilling, incineration, as well as physical and chemical recycling [5,6]. These methods usually cause secondary pollution to the environment and consume huge amounts of energy, which is not economical or environmentally friendly. Due to the improper recycling strategies and the strong mechanical properties of plastic products, serious environmental problems, such as soil pollution and the disturbance of marine ecosystems, have occurred [7]. Therefore, PET biodegradation has attracted attention as an environmentally friendly alternative, requiring milder temperatures and lower energy consumption than other recycling methods [8,9]. Additionally, the degradation monomers can easily be recycled, with the hope of converting PET into high value chemicals.

In 1977, several commercial lipases and an esterase were reported to hydrolyze various kinds of polyesters [10]. Since then, many PET hydrolases, such as lipases, cutinases and esterases, have been discovered and characterized by various microorganisms [1,11]. In 2016, *Ideonella sakaiensis* 201-F6 was isolated from a waste recycling station [12]. It was found to produce PET hydrolase (PETase) and monohydroxyethyl terephthalate (MHET) hydrolase (MHETase), which can degrade PET into intermediate products at 30 °C. Then, the structures of the two enzymes were analyzed and a series of effective enzyme modifications were carried out [13,14,15,16,17], efficiently improving the activity and stability of the two enzymes. The discovery and modification of PETase and MHETase has provided an important basis for the degradation of PET waste under ambient temperatures.

Synthetic biology and metabolic engineering strategies have been applied to the biodegradation and bioconversion of PET waste, especially in the modification of PET hydrolases, optimization of microbial chassis, and reconstruction of degradation pathways. At present, some bacteria, fungi, and marine microalgae have been reported as being good microbial chassis for PET biodegradation. The whole-cell biocatalysts have been able to achieve the initial degradation of PET. The bioconversion pathways of TPA and EG have been identified. Some microorganisms have been engineered to produce high value chemicals from PET monomers, which is an important development direction for PET upcycling. Based on these current advances, developing enhanced microbial chassis and constructing artificial microbial consortia to couple the biodegradation of PET by secreted PET hydrolases with the bioconversion of high value chemicals from monomers is a promising method to realize the circular economy of PET waste.

This review summarizes the current advances in the modified PET hydrolases, engineered microbial chassis in degrading PET, bioconversion pathways of PET monomers, and artificial microbial consortia in PET biodegradation, providing novel ideas for the future degradation of PET, and other types of polymers, by artificial microbial consortia.

## 2. PET Biodegradation

**Figure 1:**
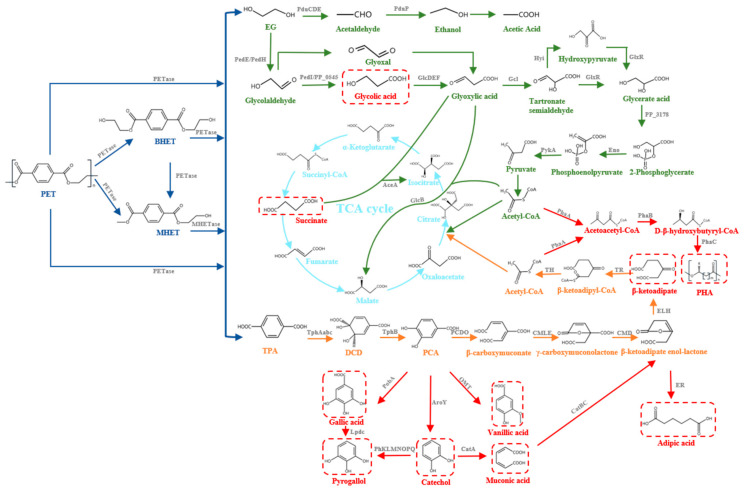
PET metabolic pathways and conversion of high value chemicals. Enzymes implicated in the pathways, PedH: quinoprotein alcohol dehydrogenase; PedE: quinoprotein alcohol dehydrogenase; PedI: aldehyde dehydrogenase family protein; PP_0545: aldehyde dehydrogenase family protein; GlcDEF: glycolate oxidase; Gcl: glyoxylate carboligase; GlxR: tartronate semialdehyde reductase; PP_3178: glycerate kinase; Eno: Enolase; PykA: Pyruvate kinase; GlcB: malate synthase; AceA: isocitrate lyase; Hyi: Hydroxypyruvate isomerase; PduCDE: propane diol dehydratase; PduP: CoA-dependent propionaldehyde dehydrogenase; TphAabc: TPA 1,2-dioxygenase; TphB: 1,2-dihydroxy-3,5-cyclohexadiene-1,4-dicarboxylate dehydrogenase; PCDO: protocatechuate 3,4-dioxygenase; CMLE: β-carboxy-cis, cis-muconate lactonizing enzyme; CMD: β-carboxymuconolactone decarboxylase; ELH: enollactone hydrolase; TR: β-ketoadipate:succinyl-CoA transferase; TH: β-ketoadipyl-CoA thiolase; PhaA: acetyl-CoA acetyltransferase; PhaB: acetoacetyl-CoA reductase; PhaC: poly(3-hydroxyalkanoate) polymerase; PobA: p-hydroxybenzoate hydroxylase; AroY: PCA decarboxylase; OMT: catechol O-methyltransferase; PhKLMNOPQ: phenol hydroxylase; CatA: a catechol 1,2-dioxygenase originating; CatBC: Muconate cycloisomerase 1/Muconolactone Delta-isomerase; ER: Enoate reductase. Steps in PET degradation; EG metabolism, TPA metabolism, TCA cycle, and high value chemical synthesis are indicated by dark blue, green, orange, light blue, and red, respectively.

During PET biodegradation, the microorganisms first adhere onto the surface of PET films and then secrete extracellular PET hydrolases, which bind to the PET films and initiate the biodegradation process [18,19]. PET hydrolases act on the ester bond of PET, hydrolyzing it into TPA and EG and generating incomplete hydrolysis products, such as MHET and Bis-(2-hydroxyethyl) terephthalate (BHET). In *I. sakaiensis* 201-F6, MHET can be further hydrolyzed into TPA and EG under the action of MHETase [12]. It was reported that MHETase has a hydrolysis activity against the termini-generated PET film, demonstrating the exo-PETase function of the enzyme [20]. PET hydrolases can further hydrolyze BHET to produce MHET, TPA, and EG [12]. The products TPA and EG can be used by different microorganisms and be further metabolized into the tricarboxylic acid cycle (TCA cycle) [21,22,23,24,25,26,27,28,29]. Additionally, these intermediate and final products of PET biodegradation have been identified as competitive inhibitors of PET hydrolases [30,31] (Figure 1).

### 2.1. Engineered PET Hydrolases

The hydrolases, including lipases [31,32,33,34], cutinases [35,36,37,38,39,40,41,42], esterases [43,44,45,46], PETase [12] and MHETase [12], that can degrade PET have been identified. Among them, lipases have the lowest hydrolysis activity of PET mainly because their catalytic centers are covered by lid structures, which limits the hydrolases’ contact and catalysis with the substrate PET. Cutinases always have a strong PET hydrolysis ability due to their large substrate binding pockets without lid structures, which is conducive to the combination of PET with their active centers. However, cutinases usually degrade PET at high temperatures (50–70 °C), while PETase and MHETase can efficiently and specifically hydrolyze PET at 30 °C [12]. The discovery of PETase and MHETase is helpful in achieving the high efficiency biodegradation of PET at ambient temperatures. At present, the structures of these two enzymes have been studied extensively, and more high activity hydrolases variants have appeared.

The PET hydrolases identified in nature always have poor stability, low activity, and low expression levels, which limit their large-scale industrial application. A series of strategies that could enhance the catalytic activity of PET hydrolases have been proposed [13] (Table 1).

One strategy is to engineer the binding pocket, which can improve the specificity of the PET hydrolases and increase the effective adsorption of enzymes and substrates [15,47,48,49]. Our laboratory previously focused on six key amino acids near the binding of PETase to the substrate and conducted site-directed mutations. The R61A, L88F, and I179F mutants were successfully screened, and the enzyme activity increased 1.4-fold, 2.1-fold, and 2.5-fold, respectively, in comparison to wild-type PETase [50]. Silva et al. [51] modified the cutinase from *Thermobifida fusca*_0883 by site-directed mutagenesis and constructed a single mutation Ile218Ala and a double mutation Q132A/T101A, which expanded the catalytic space and improved the efficiency of the PET biodegradation. Chen et al. [52] identified the unique amino acids S214 and I218 through the structural analysis of PETase and noted that they are associated with W185 wobbling and β6-β7 loop flexibility. This research is helpful in designing PETase mutants that increase the flexibility of the substrate binding pocket.

Some studies focused on using enzyme engineering strategies to improve the stability of the PET hydrolases to improve PET biodegradation efficiency [53,54]. Methods such as adding Ca^2+^ or Mg^2+^ [38,55], introducing a disulfide bond and salt bridge [56,57], and glycosylation have all been proven to improve the stability of PET hydrolases. Researchers added disulfide bonds to improve the thermal stability of leaf-branch compost cutinase (LCC) and performed site-directed mutations on hot amino acids near the substrate binding to obtain the combined mutation F243I/D238C/S283C/Y127G (ICCG) [53]. Finally, 90% of shredded PET plastic bottles were degraded at 72 °C for 10 h, which is by far the most efficient PET hydrolase [53].

Additionally, increasing the substrate accessibility for PET hydrolases by engineering the PET hydrolases has also been widely studied [58,59,60]. It is reported that the fusion expression of Thc_Cut1 from *Thermobifida cellulosilytica* and hydrophobins (HFB4 and HFB7) from *Trichoderma reesei* can increase the hydrolysis effect of PET by more than 16 times, while a mixture of the enzyme and the hydrophobins led to only a 4-fold increase at most [61].

The intermediate and final products of PET biodegradation, such as Bis-(2-hydroxyethyl) terephthalate (BHET), monohydroxyethyl terephthalate (MHET), TPA, and EG, are all competitive inhibitors of the PET hydrolases [30]. Therefore, the mixtures of hydrolases that act synergistically or protein engineering strategies that reduce the interaction between the enzymes and products are effective methods for solving the inhibition [62,63,64].

In addition, other strategies have been studied to increase the activity of the enzymes and enhance the biodegradation of PET [36,38,57,59,65].

### 2.2. Engineered PET Biodegradation Chassis

Most of the microorganisms identified that are capable of secreting PET hydrolases are non-model microorganisms and they are difficult to genetically engineer due to their complex genetic background. In addition, the expression level of the PET hydrolases from wild strains is insufficient to satisfy the demand for large-scale degradation. Therefore, it is necessary to develop recombinant expression systems using model microorganisms to express the PET hydrolases efficiently. PET is a high molecular polymer that is polymerized from TPA and EG and cannot enter cells, so in vitro enzymatic degradation of PET has been studied extensively. Owing to the purification and preparation process of PET hydrolases being time-consuming and cost-intensive, the efficient expression PET hydrolases extracellularly for practical applications is necessary [76,77].

At present, some microbial chassis such as bacteria, fungi, and marine microalgae have been applied to the secretion and expression of PET hydrolases, which have been studied and proven to be promising chassis to degrade PET (Table 2). Several whole-cell biocatalysts have been designed to degrade PET, which are able to not only avoid the complicated steps of enzyme purification but also be reused in multi-step reactions, in comparison to the free enzyme-based approach [78,79]. Additionally, the difficulty of the reduced activity of the enzymes, or even enzymes being inactivated, under the influence of environmental factors has been solved. The following is a summary of several microbial chassis that are suitable for PET biodegradation.

#### 2.2.1. Bacteria

##### Escherichia coli

*E. coli* is an important model microorganism for the production of recombinant proteins due to its clear genetic background, simple growth conditions, and its advantages in high density cultivation [89]. In recent years, with the continuous discovery of PET hydrolases, more and more enzymes have achieved the heterologous expression in *E. coli* [12,14,16,50,53,69]. PET hydrolases heterologously expressed in *E. coli* have been summarized [76] and it is helpful in further analyzing the crystal structures of these enzymes and explore the degradation mechanism for PET.

Recent studies have shown that engineered *E. coli* can be used as a whole-cell biocatalyst for PET biodegradation. Selecting the optimal signal peptide is a common strategy used to improve the section of heterologous PET hydrolases. A study tested the effects of Sec-dependent and SRP-dependent signal peptides from *E. coli* in secreting PETase, and successfully produced 6.2 mg/L PETase by fusing SP_LamB_ and PETase [80]. Some other research improved the expression titer and enzymatic activity by modifying the signal peptide. An evolved signal peptide PelB (G58A) obtained by random mutagenesis was successfully used to express heterologous PETase in *E. coli* and enabled up to 1.7-fold higher PETase secretion [81]. An enhancer of signal peptides B1 (MERACVAV) was studied to mediate the excretion of PETase, and finally, the excretion efficiency of PETase mediated by B1PelB demonstrated a 62-fold increase over that of PelB [82].

##### Bacillus subtilis

Gram-positive *B. subtilis* has the advantages of high secretion capacity, fast growth, and the lack of an outer membrane, and it is regarded as an excellent microbial chassis for secreting heterologous proteins compared to *E. coli*, which usually forms an inclusion body [90,91]. Additionally, *B. subtilis* has a strong resistance to harsh environments and it has been used to secrete proteins that can degrade many pollutants, which is why it is considered to be a promising microbial chassis for biodegradation [92,93].

In terms of PET biodegradation, *B. subtilis* has been engineered to secrete PET hydrolases. It is reported that PETase was successfully secreted into the medium by *B. subtilis* 168 under the direction of its native signal peptide (SP_PETase_). SP_PETase_ is predicted to be a twin-arginine signal peptide, and the inactivation of twin-arginine translocation (Tat) complexes improved the secretion amount of PETase 3.8-fold [83]. Another two PET hydrolases (BhrPETase and LCC) were also expressed in *B. subtilis*, and the expression titer of BhrPETase and LCC reached 0.66 g/L and 0.89 g/L in an engineered chaperone-overexpression of *B. subtilis*, respectively [42]. Additionally, the combinations of signal peptides and promoters were optimized to promote the expression of PETase in *B. subtilis* WB600, and the combination of the signal peptide SP_amy_ and the weak promoter P43 was proved to be best [84].

##### Thermophilic Bacteria

Most of the hydrolases capable of degrading PET, including lipases, cutinases and esterases, have higher enzymatic activity at higher temperatures, while the optimal growth temperature of most model microorganisms that can produce heterologous PET hydrolases is usually 30–40 °C. Whole-cell biocatalyst is not compatible with some PET hydrolases that are only functional at high temperatures [94]. Therefore, a thermophilic expression system is necessary to improve the efficiency of PET biodegradation [36,95]. Most thermophilic microorganisms are usually difficult to genetically engineer except for *Clostridium thermocellum*, which has a mature genetic manipulation platform [94]. *C. thermocellum* has been engineered for lignocellulose bioconversion [96] and biofuel production [97], which is why it is regarded as a potential microbial chassis for the biodegradation of PET.

LCC has been successfully obtained from an engineered *C. thermocellum*. This engineered whole-cell biocatalyst realized a high level expression of LCC and more than 60% of a commercial PET film was converted into soluble monomers at 60 °C after 14 days [85]. This thermophilic whole-cell degradation system has the advantage of simultaneous enzyme production and PET degradation compared to only using free enzymes, which is why it is a promising strategy to degrade PET using other high temperature hydrolases [98,99]. In addition to the thermophilic whole-cell degradation system, an alkali-tolerant whole-cell catalytic system has also been reported [100,101,102].

#### 2.2.2. Fungi

In addition to bacteria, the potential of some yeasts, including *Pichia pastoris* and *Yarrowia lipolytica*, being used in PET biodegradation has been studied. *P. pastoris*, with a great secretion expression system and scalable fermentation capability, has become a common strain for protein production in industrial applications. Researchers have expressed BurPL (H344S/F348I) and PETase in *P. pastoris* and *E. coli* and noted that both enzymes produced from *P. pastoris* showed higher activity than that expressed in *E. coli* because of the protein half-life protection mechanism of *P. pastoris* [52,103]. A whole-cell biocatalyst was developed by displaying PETase on the surface of *P. pastoris* and the enzymatic activity of PETase increased 36-fold towards a highly crystalline PET in comparison to that of purified PETase [86]. Additionally, this whole-cell biocatalyst can be reused seven times without obvious activity loss, which is helpful in developing other whole-cell biocatalysts for PET biodegradation [86]. Considering the ability of *P. pastoris* to perform N-linked glycosylation, some researchers studied the effects of glycosylation on the LCC expressed in *P. pastoris* and found that the kinetic stability and activity of LCC were both improved [35]. *Y. lipolytica* is also a great microbial chassis for bioremediation [104]. Researchers isolated *Y. lipolytica* IMUFRJ 50682 with the ability to convert PET into MHET and verified that the PET monomers may act as inducers in the process of lipase production [105], which showed that *Y. lipolytica* is a potential microbial chassis for PET biodegradation. Other research expressed PETase in *Y. lipolytica* Po1f with a signal peptide from lipase and confirmed that the engineered strain could hydrolyze BHET and PET powder into the monomers [106]. Surface display systems and whole-cell biocatalysts provide novel ideas and strategies for achieving the high efficiency expression of PET hydrolases and promoting PET biodegradation [77,107,108]. Yeasts, together with efficient genetic tools, have been used as great microbial chassis for biodegradation and bioconversion [109].

#### 2.2.3. Marine Microalgae

At present, the existing native and engineered microbial chassis that are capable of producing PET hydrolases are usually difficult to adapt to the complexity of the marine environment and produce much PET waste. Recently, some marine microalgae have been used as chassis for PET biodegradation [110]. A photosynthetic microalga *Phaeodactylum tricornutum* has been reported as being engineered as a chassis capable of secreting a PETase mutant into the culture medium, and the recombinant PETase was able to efficiently degrade different substrates, including PET films, poly (ethylene terephthalateco-1,4-cylclohexylenedimethylene terephthalate) (PETG) film, and shredded PET, at 30 °C or even at mesophilic temperatures (21 °C) [87]. Additionally, *Chlamydomonas reinhardtii*, the green algae, was also successfully engineered to produce PETase with degrading activity, and the chemical and morphological changes appeared on the PET films after 4 weeks of culture [88]. As environmentally friendly chassis for the biodegradation of PET waste in a saltwater-based environment, marine microalgae have the potential for future biotechnological applications in the degradation of PET polluted seawater [87].

## 3. Metabolism and Bioconversion of PET Monomers

It is reported that *Acetobacterium woodii*, *Pseudomonas sp.,* and *E. coli* have ability to utilize EG. In *A. woodii*, EG can be utilized by an acetaldehyde/ethanol pathway while it is consumed by a glyoxylic acid pathway in *Pseudomonas sp.* and *E. coli*. TPA can be also utilized by some bacteria, such as *Rhodococcus sp*., etc. Additionally, a series of metabolic engineering strategies have realized the bioconversion of EG and TPA into high value chemicals, such as glycolic acid, PHA, gallic acid, vanillic acid, and β-ketoadipic acid. The metabolism and bioconversion pathways of the PET monomers, TPA and EG, are shown in Figure 1.

### 3.1. Metabolism of EG

At present, two naturally existing pathways, including the acetaldehyde/ethanol pathway and glyoxylic acid pathway for the utilization of EG by microorganisms, have been reported (Figure 1, green pathway). The use of EG is not commonly reported in metabolic engineering of model microorganisms, except for *E. coli*.

#### 3.1.1. Acetaldehyde/Ethanol Pathway

The acetogenic bacterium *A. woodii* can use EG as the sole carbon source for growth, and the EG metabolic pathway has been identified [25]. EG is dehydrated to acetaldehyde, catalyzed by the propane diol dehydratase (PduCDE), then further converted into ethanol and acetyl coenzyme A (acetyl-CoA), catalyzed by CoA-dependent propionaldehyde dehydrogenase (PduP) [111]. PduCDE and PduP are both encoded by the Pdu gene cluster [111]. Acetyl-CoA and a part of the ethanol are converted into acetic acid, and this process is accompanied by the production of adenosine triphosphate (ATP) and nicotinamide adenine dinucleotide (NADH) [111]. The reducing equivalents of the ethanol oxidation are recycled through the reduction of carbon dioxide (CO_2_) into acetate in the Wood–Ljungdahl pathway [25]. The acetaldehyde/ethanol pathway is commonly found in some Clostridium species and a few other anaerobic organisms because the enzymes that catalyze EG are oxygen sensitive. Additionally, Dragan et al. [25] and Nilanjan et al. [112] proved that the enzymes for EG utilization were encapsulated in bacterial microcompartments.

#### 3.1.2. Glyoxylic Acid Pathway

Glyoxylic Acid Pathway in *Pseudomonas sp.*

In *Pseudomonas aeruginosa* and *Pseudomonas putida*, EG is converted into glyoxylic acid under the action of dehydrogenase and finally enters the TCA cycle through different routes [21,22,23,113]. At present, the metabolic pathway of EG in *P. putida* KT2440 is the most widely studied. The metabolism pathways in utilizing EG have been well demonstrated in *P. putida* KT2440, in comparison to other bacteria, and related enzymes have been identified. In *P. putida* KT2440, two functionally redundant periplasmic quinoproteins, PedE and PedH, catalyze EG into glycolaldehyde [114]. PedE and PedH are both pyrroloquinoline quinone-dependent alcohol dehydrogenases (PQQ-ADHs), and their expression depend on Ca^2+^ and lanthanide metal ions, respectively [114]. Once glycolaldehyde is produced, the two cytoplasmic aldehyde dehydrogenases, PP_0545 and PedI, catalyze it into glycolic acid, and glyoxylic acid is further generated via the membrane anchored oxidase GlcDEF. The glyoxylic acid is converted into acetyl-CoA and enters the TCA cycle to be catalyzed by a series of enzymes [115]. Additionally, there are another two alternative pathways to convert glyoxylic acid, one of which is catalyzed by isocitrate lyase (AceA) and glyoxylic acid can condense with succinic acid to form isocitrate. The other is catalyzed by malate synthase (GlcB) and glyoxylic acid condenses with acetyl-CoA to form malic acid. However, due to the removal of CO_2_ and the restriction of the amount of acetyl-CoA, *P. putida* KT2440 cannot use EG as the sole carbon source for growth [115]. Researchers engineered *P. putida* KT2440 by overexpressing glycolate oxidase to remove the glycolate metabolic bottleneck and produced an engineered strain that can efficiently metabolize EG [115]. After that, mutants of *P. putida* KT2440 that utilize EG as the sole carbon source were isolated through adaptive laboratory evolution, and the metabolism and regulation mechanism of EG in *P. putida* KT2440 was further clarified [116]. *P. putida* JM37 was reported to be able to use EG as the sole carbon source for growth because there is another pathway to use glyoxylic acid compared to *P. putida* KT2440. Glyoxylic acid is converted into tartrate semialdehyde under the catalysis of glyoxylate carboxylase (Gcl) and then tartrate semialdehyde is converted into glycerate acid, catalyzed by hydroxypyruvate isomerase (Hyi) and tartrate semialdehyde reductase (GlxR). Glycerate acid can be further converted into 2-phosphoglycerate and enter the TCA cycle [117].

Glyoxylic Acid Pathway in *E. coli*

Wild-type *E. coli* cannot use EG as the sole carbon source for growth [118]. In 1983, researchers first reported an *E. coli* strain capable of using EG as the sole carbon source from the propylene glycol using mutants. They identified the increased activities of propanediol oxidoreductase, glycolaldehyde dehydrogenase, and glycolate oxidase in the mutants [118]. Based on this discovery, researchers began to design and construct engineered *E. coli* that could use EG to convert PET monomers into high value chemicals.

EG is assimilated and oxidized into glycolaldehyde and, subsequently, into glycolic acid under the catalysis of 1,2-propanediol oxidoreductase mutant (fucO) and glycolaldehyde dehydrogenase (aldA), respectively. Glycolic acid can be metabolized into glyoxylic acid by glycolate dehydrogenase (GlcDEF) [26]. Similar to *P. putida*, glyoxylic acid is further condensed into acetyl-CoA by the linear glycerate pathway or converted into isocitrate and malate catalyzed by AceA and GlcB, respectively. An engineered *E. coli* can take EG as the sole carbon source to produce glycolate by expressing fucO mutant (I7L/L8V) and aldA. Experiments identified that oxygen concentration was as an important metabolic valve, and flux to 2-phosphoglycerate was the primary route in the assimilation of EG as a substrate combining modeling [113,119]. Additionally, EG can be efficiently utilized in *E. coli* by optimizing the gene expression (fucO and aldA) and adding a growth medium with a low concentration of glycerol or a mixture of amino acids [26]. Although *E. coli* MG1655 contains the endogenous glyoxylic acid metabolism pathway, the EG-utilizing ability of the engineered *E. coli* still needs to be improved [119]. Introducing a heterologous pathway or unblocking the rate-limiting steps of the EG metabolic pathway in *E. coli* may further enhance the assimilation of EG. *E. coli* has a clear genetic background and simple genetic operations compared to other bacteria, so it is easier to engineer it to transform EG into high value chemicals.

### 3.2. Bioconversion of EG to High Value Chemicals

EG is one of the cheap raw materials for glycolic acid production through incomplete oxidation. Several wild microorganisms, including *Pichia naganishii* [23], *Rhodotorula sp.* [23], *Burkholderia sp.* [120], *Gluconobacter oxydans* [24], and *Hansenula sp.* [121], have been reported to produce glycolic acid from EG. Among these microorganisms, *G. oxydans* has been extensively studied due to its high titer of glycolic acid from EG. It is reported that the overexpression of membrane-bound alcohol dehydrogenase (mADH) in *G. oxydans* DSM 2003 accelerated cell growth, and 113.8 g/L of glycolic acid was accumulated with a molar yield of 92.9% within 45 h [122]. Two genes encoding recombinant cytosolic oxidoreductases (gox0313 and gox0646) from *G. oxydans* were heterologously expressed in *E. coli* and the resulting proteins were purified and characterized [123]. In addition to *G. oxydans*, engineered *E. coli* has potential in producing glycolic acid from EG, and 10.4 g/L of glycolic acid was produced from EG after 112 h in a fed-batch bioreactor using a series of oxygen-based strategies [26,118].

EG can also be used to produce polyhydroxyalkanoate (PHA) by *P. putida* under nitrogen-limiting conditions [124]. An engineered strain *P. putida* KT2440 realized the conversion of EG into mcl-PHAs [115,124] and some metabolic engineering strategies were developed to enhance medium chain length polyhydroxyalkanoates (mcl-PHAs) production in *P. putida* [125,126,127]. mcl-PHAs can be upgraded into chemical precursors and fuels via a straightforward catalytic process [128].

### 3.3. Metabolism of TPA

It was reported that *Comamonas sp*. [27], *Delftia tsuruhatensis* [129], *Comamonas testosterone* [130], and *Rhodococcus sp*. [28] can use TPA as the sole carbon source for their growth (Figure 1, orange pathway). In these bacteria, TPA enters the cell via the TPA transporters [29]. Generally, TPA can be transformed into 1,6-dihydroxycyclohexa-2,4-diene-dicarboxylate (DCD) under the catalysis of TPA dioxygenase (TphAabc), and DCD is further oxidized by 1,2-dihydroxy-3,5-cyclohexadiene-1,4-dicarboxylate dehydrogenase (TphB) to form protocatechuate (PCA) [130,131,132,133]. The genes responsible for these reactions have been characterized [27,28,129,130]. *Comamonas sp*. E6 also contains the extra gene TphC, which encodes a permease involved in TPA uptake using the tripartite aromatic acid transporter [29]. There are three main pathways for the metabolism of PCA, the ortho-, meta-, and para-cleavage pathways, which are catalyzed by 3,4-dioxygenase (PCDO), 4,5-dioxygenase, and 2,3-dioxygenase, respectively [134,135,136,137]. At present, the ortho-cleavage pathway is the most extensively studied, and PCA is converted into β-carboxymuconate under the catalysis of protocatechuate 3,4-dioxygenase (PCDO), is finally converted into acetyl-CoA, and enters the TCA cycle [134,138].

### 3.4. Bioconversion of TPA to High Value Chemicals

It has been demonstrated that the PET monomer TPA is suitable for the biosynthesis of several high value chemicals, such as gallic acid, pyrogallol, catechol, muconic acid, vanillic acid, catechol, adipic acid, PHA, and β-ketoadipic acid [106,139,140,141,142,143]. Since PCA is an important precursor in producing a series of high value aromatic chemicals, the key to the bioconversion of TPA is the acquisition of PCA. By the heterologous expression of TPA, 1,2-dioxygenase (TphAabc), and DCD dehydrogenase (TphB) from *Comamonas sp.*, *E. coli* was engineered to utilize TPA and produced PCA [133]. Further heterologous expression of different enzymes produced gallic acid, pyrogallol, catechol, muconic acid and vanillic acid from PCA in *E. coli* [133]. Additionally, a novel pathway for the direct upcycling of TPA into the value-added small molecule vanillin was reported in engineered *E. coli* and the conversion efficiency reached 79% [143].

PHA can also be produced from TPA. Researchers have isolated *P. putida* GO16 and *P. putida* GO19 from a PET bottle processing plant and proved their ability to convert TPA into PHA at a maximal rate of approximately 8.4 mg·L^−1^·h^−1^ for 12 h [144]. Recently, researchers engineered *Pseudomonas umsongensis* GO16 to convert PET into two types of bioplastics, PHA and a novel bio-based poly (amide urethane) (bio-PU), and further achieved the secretion of hydroxyalkanoyloxy alkanoates (HAAs) by introducing the HAA synthesis module into the engineered strain [145]. Poly-(R)-3-hydroxybutyrate (PHB), the first PHA discovered, has also been produced from PET through the heterologous expression of the phbCAB operon from *Ralstonia eutropha* in *Pseudomonas stutzeri* [106]. Due to the same synthetic precursors of rhamnolipids and PHA, many microorganisms capable of converting PET into PHA also have the potential to synthesize rhamnolipids [144]. The conversion of PET into biodegradable plastics is a clean and cost-effective way to generate a great market in PET recycling [146].

As for producing β-ketoadipic acid from TPA, four sequential metabolic engineering efforts in *P. putida* KT2440 were performed to directly convert BHET into β-ketoadipic acid [139]. The engineered *P. putida* is able to not only degrade BHET into TPA and EG, but also convert TPA into 15.1 g/L of β-ketoadipic acid at 76% molar yield in bioreactors [139]. β-ketoadipic can be further polymerized into a nylon-6,6 analog, or other products [147].

PET waste is depolymerized by microorganisms in nature and converted into CO_2_ and water, which causes serious resource loss and carbon emissions. Therefore, utilizing PET and its monomers to produce high value chemicals provides a new solution for upgrading and recycling PET and other plastics waste [148].

## 4. Microbial Consortia in PET Biodegradation

Artificial microbial consortia that simulate natural microbial consortia to complete complex biological processes have become an important research topic in synthetic biology [86,89,149,150]. It is important to explore the potential and reprogram the functionality of microbial consortium members for specific approaches, especially for the bioconversion of contaminants [151,152]. Previous studies have highlighted the potential for biodegradation and bioconversion using artificial microbial consortia [153,154,155]. Artificial microbial consortia have been used to degrade hydrocarbons [153], organophosphorus pesticides [156], polyaromatic hydrocarbon pollutants [157], and aryl organophosphate flame retardants (aryl-OPFRs) [158] and improve the desulfurization of petroleum sulfides [159]. In addition, some artificial microbial consortia have been constructed in degrading plastic waste, such as polyurethane (PU) [154], polyethylene (PE) [160,161], polypropylene (PP) [162], and polyvinyl chloride (PVC) [163]. These results highlight the potential of artificial microbial consortia in PET biodegradation.

There are specific advantages in PET biodegradation by artificial microbial consortia compared to pure culture: (i) the synergies of different enzymatic systems and combination of the metabolic pathways of various microorganisms can relieve the inhibition of degradation products and improve degradation rate; (ii) PET biodegradation and bioconversion can be realized simultaneously by different microorganisms; and (iii) the construction of artificial microbial consortia is more efficient and time-saving than other metabolic engineering strategies [164]. Therefore, the application of artificial microbial consortia in the biodegradation and bioconversion of PET is regarded as a promising method to realize the circular economy of PET waste.

### 4.1. Natural Microbial Consortia in PET Biodegradation

At present, most microbial consortia that are capable of degrading PET are natural microbial consortia. Researchers isolated a consortium, including three *Pseudomonas sp.* And two *Bacillus sp*., that can reduce the granular PET and they identified that a 100 mg PET granule weighed 3.15 mg less when with the consortium for six weeks, which suggests that the strains can act synergistically to degrade PET [165]. After that, researchers screened for lipase activity associated with PET biodegradation and proved that the secreted enzymes extracted from the consortium could fully convert BHET into TPA and EG [166]. Another study reported a consortium, including *Bacillus cereus* SEHD031MH and *Agromyces mediolanus* PNP3, from activated sludge. It noted that the consortium could use PET microplastics (MPs) as the sole carbon source and degrade 17% of PET MPs at 30 °C of the course of 168 days [167]. Additionally, Oberbeckmann et al. [168] analyzed the influence of different seasons, geographic locations, seawater, and substrate material types on the microbial consortia of using single-use PET bottles at multiple stations in the North Sea.

Most PET hydrolases that have been previously reported, such as cutinases, lipases, and esterases, can only accomplish the limited degradation of PET. In 2016, Yoshida et al. [12] successfully isolated a microbial consortium No. 46 that degraded amorphous PET from a waste recycling station completely at ambient temperature. Then, a bacterium capable of degrading and assimilating PET named *I. sakaiensis* 201-F6 was isolated from No. 46 consortium. It could produce PETase and MHETase to degrade PET, which provided a new direction for the biodegradation of PET under ambient temperatures [4,12].

Marine microbial consortia can colonize PET, form biofilms on its surface, and finally modify its chemical structure [169,170,171,172]. A study demonstrated for the first time that hydrocarbon-degrading marine consortia enriched on tetradecane and diesel have the potential to degrade PET and cause major alterations to the surface structure and hydrophobicity of PET films [173].

### 4.2. Artificial Microbial Consortia in PET Biodegradation

At present, there are few studies on the construction of artificial microbial consortia to degrade PET. A distinct three-consortium named CAS6 was isolated from an ocean bay and it can make PET films lose sharp morphology, compared to controls after 4 weeks [174]. Then, three bacteria (*Exiguobacterium sp.*, *Halomonas sp.,* and *Ochrobactrum sp.*) were identified from CAS6 and formed a stable artificial three-microbial consortium in a 1:1:1 ratio to efficiently degrade PET films. PET films were fully degraded into small pieces after 2 weeks of incubation by the three-microbial consortium [174]. Pan et al. [106] designed an engineered *Y. lipolytica* to secrete PETase in order to degrade PET and an engineered *Pseudomonas stutzerithe* to convert TPA into PHB. They constructed a microbial consortium with two engineered strains to achieve the conversion of BHET into PHB over the course of 54 h. This was the first attempt at performing the enzymatic hydrolysis of PET and the bioconversion of TPA simultaneously. Although PHB could not be synthesized directly from PET because of the low hydrolyzing efficiency of PETase, it demonstrated the possibility of artificial microbial consortia achieving the simultaneous degradation and upcycling of PET [106]. Our laboratory has constructed a four-species microbial consortium composed of two metabolically engineered *B. subtilis*, *Rhodococcus jostii* and *P. putida* to degrade PET, and the weight loss of PET film reached 23.2% under ambient temperature [149]. The artificial microbial consortium successfully relieved the metabolic inhibition of TPA and EG, and effectively improved the degradation rate [149].

### 4.3. Prospect of PET Biodegradation by Artificial Microbial Consortia

**Figure 2:**
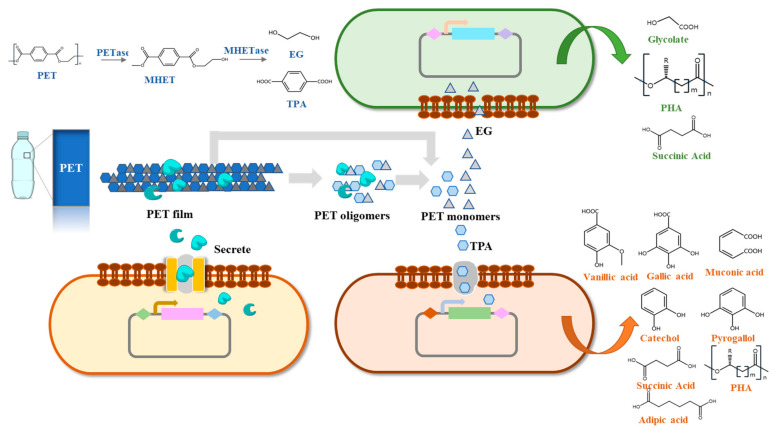
Artificial microbial consortia for PET biodegradation and bioconversion.

The artificial microbial consortia are expected to effectively solve the problems in the PET biodegradation process and improve the degradation efficiency. During the biodegradation of PET, the intermediate and final products, such as BHET, MHET, TPA, and EG, have been identified as competitive inhibitors of PET hydrolases [30,31,115]. Constructing artificial microbial consortia can relieve the inhibition of metabolites and promote biodegradation, which has been demonstrated by a two-microbial consortium for corn fiber conversion [175]. By combining the PET-degrading module and the monomer-converting modules to construct an artificial microbial consortium, we speculate that the inhibitory effect of TPA and other substances can be relieved (Figure 2). In addition, only using a single bacterium to degrade PET and convert the degradation products will increase its metabolic burden, while an artificial microbial consortium can solve that problem. A three-microbial consortium, including three engineered *E. coli,* was constructed to reduce the metabolic burden and synthesize rosmarinic acid [176]. Another three-microbial consortium was constructed to produce short chain fatty acids from lignocellulose and it can reduce the metabolic burden and perform multiple tasks [177].

In view of the current problems of PET biodegradation, the artificial microbial consortium is an important metabolic strategy to solve them. Artificial microbial consortia can be used to perform more complex tasks in a more complex environment [178,179,180,181]. Constructing a PET-degrading module, an EG-converting module, and a TPA-converting module to perform different functions can accelerate the degradation of PET and realize the complete conversion of it [149]. Artificial microbial consortia can couple the depolymerization of PET by secreted enzymes with the biosynthesis of high value chemicals from monomers, which is a promising strategy in realizing the circular economy of PET waste [106].

## 5. Conclusions

This review summarized the current advances of PET biodegradation and bioconversion from the four aspects of engineered enzymes, chassis, pathways, and consortia, which provide a basis for the construction of artificial microbial consortia to convert PET into high value chemicals. Artificial microbial consortium is a promising strategy in realizing the circular economy of PET waste. On the one hand, the artificial microbial consortia are expected to effectively release the competitive inhibition of monomers in the PET biodegradation and improve the degradation efficiency. On the other hand, the artificial microbial consortia can couple the biodegradation of PET with the bioconversion of high value chemicals from monomers to realize circular economy and sustainability. Owing to the recent advancements in synthetic biology and metabolic engineering, it has now become possible to rationally design and create artificial microbial consortia with a superior metabolic efficiency to degrade PET and convert it into high value chemicals in one step.

## Figures and Tables

**Table 1:** Engineered hydrolases for PET biodegradation.

Strategy	Hydrolase	Source	Variant	Substract	pH	Temperature	Time	Effect ^1^	References
Engineering the key binding sites	MHETase	*Ideonella sakaiensis* 201-F6	F424Q/F424N	BHET	7.5	30 °C	19.25 h	Turnover rate: >0.12 s^−1^	[14]
	MHETase	*Ideonella sakaiensis* 201-F6	W397A, F415H, H488A	MHET	7.5	30 °C	19.25 h	Turnover rates: 2.2-, 1.6-, and 1.15-fold, respectively	[14]
	MHETase	*Ideonella sakaiensis* 201-F6	S416A/F424N, R411A/S419G/F424N	BHET	7.5	30 °C	19.25 h	Turnover rate: >0.12 s^−1^ (120-fold)	[14]
	PETase	*Ideonella sakaiensis* 201-F6	S238F/W159H	PET of ~15% crystallinity	7.2	30 °C	96 h	Crystallinity loss: 4.13%; TPA release rate: 1.2-fold	[15]
	PETase	*Ideonella sakaiensis* 201-F6	S238F/W159H	Semi-aromatic polyester PEF	7.2	30 °C	96 h	Crystallinity loss: 1.3%; FDCA release rate: 1.2-fold	[15]
	MHETase	*Ideonella sakaiensis* 201-F6	F424N, F424V, F424I, R411K, R411K/F424N, R411K/F424V, R411K/F424I, R411K/S416A/F424I	BHET	8.0	30 °C	4 h	Activity: 3.9-, 3.0-, 3.4-, 1.7-, 8.7-, 10.5-, 11.1-, and 15.3-fold, respectively	[20]
	MHETase	*Ideonella sakaiensis* 201-F6	R411K/S416A/F424I	PETase^S121A/D186H/R280A^-treated PET films	8.0	30 °C	72 h	Activity: 2-fold	[20]
	PETase	*Ideonella sakaiensis* 201-F6	Y58A, W130A, W130H, A180I, S185H	Drinking bottle	9.0	30 °C	48 h	Activity: 3.5-, 1.9-, 3.3-, 2.0-, and 2.3-fold, respectively	[49]
	PETase	*Ideonella sakaiensis* 201-F6	R61A, L88F, I179F, Y58A	Amorphous PET films	8.5	30 °C	48 h	Activity: 1.4-, 2.1-, and 2.5-fold, respectively	[50]
	Tfu_0883	*Thermobifida fusca*	Q132A/T101A, I218A	PET fabric (100% polyester)	7.5	60 °C	48 h	Productivity of TPA: 19.3 ± 0.1 mM (1.6-fold increase) and 15.4 ± 0.1 mM (1.3-fold increase), respectively	[51]
	BurPL	*Burkholderiales* bacterium RIFCSPLOWO2_02_FULL_57_36)	H344S/F348I	PET powder/Goodfellow PET	9.0	35 °C	18 h	Productivity of the MHET and TPA: ~3-fold increase	[52]
	PbPL	*Polyangium brachysporum*	H216S/F220I	PET powder/Goodfellow PET	9.0	40 °C	18 h	Productivity of the MHET and TPA: ~10-fold increase	[52]
	CtPL	*Caldimonas taiwanensis*	H210S/F214I	PET powder	9.0	60 °C	18 h	Productivity of the MHET: 23.12 ± 2.14 μM; Productivity of the TPA: 6.74 ± 1.21 μM	[52]
	PET2	Metagenomics analysis	H229S/F233I	PET powder	9.0	50 °C	18 h	Productivity of the TPA: 30.31 ± 0.13 μM	[52]
	LCC	Leaf-branch compost	H218S/F222I	PET powder	9.0	60 °C	18 h	Productivity of the MHET: ~900 μM; Productivity of the TPA: ~450 μM	[52]
	TfCut2	*Thermobifida fusca*22	H185S/F189I	PET powder	9.0	60 °C	18 h	Productivity of the MHET: ~120 μM; Productivity of the TPA: ~80 μM	[52]
	FsC	*Fusarium solani pisi*	L81A	PET fibers	7.5	37 °C	24 h	Activity: 5-fold increase	[66]
	Cut190	*Saccharomonospora viridis* AHK190	I224A/Q138A	BHET	8.2	37 °C		Catalytic activity: 150 ± 0.2 s^−1^	[67]
	PETase	*Ideonella sakaiensis* 201-F6	R280A	Commercial PET films	9.0	30 °C	18 h/36 h	Activity: increased by 22.4% in 18 h and 32.4% in 36 h	[68]
	PE-H	*Pseudomonas aestusnigri*	Y250S	Amorphous PET films	7.4	30 °C	48 h	Productivity of MHET: >5 mg/L	[69]
	PE-H	*Pseudomonas aestusnigri*	Y250S	Commercial single use bottle	7.4	30 °C	48 h	Productivity of MHET: >0.12 mg/L	[69]
Improving the stability	LCC	Leaf-branch compost	LCC-G	PET films of 7% crystallinity	8.0	70 °C	48 h	Weight loss: ~95%	[35]
	Cut190	*Saccharomonospora viridis* AHK190	S226P/R228S	Amorphous PET films	8.2	63 °C	3 d	Weight loss: 14% for PET-GF and 27% for PET-S	[37]
	TfCut2	*Thermobifida fusca*	D174R, D204R, E253R	Low crystallinity PET films	8.5	65 °C	48 h	Weight loss: 6.9 ± 0.0%, 11.3 ± 0.3%, and 10.1 ± 0.3%, respectively	[38]
	LCC	Leaf-branch compost	F243I/D238C/S283C/Y127G	Post-consumer colored-flake PET waste	8.0	72 °C	9.3 h	Degradation rate: 90%; Productivity of TPA: 16.7 g·L^−1^·h^−1^	[53]
	LCC	Leaf-branch compost	F243W/D238C/S283C/Y127G	Post-consumer colored-flake PET waste	8.0	72 °C	10.5 h	Degradation rate: 90%; Productivity of TPA: 16.7 g·L^−1^·h^−1^	[53]
	PETase	*Ideonella sakaiensis* 201-F6	S214H/I168R/W159H/S188Q/R280A/A180/G165A/Q119Y/L117/T140D	PET films of 49.2% crystallinity	9.0	40 °C	10 d	Activity: 400-fold	[54]
	Est119	*Thermobifida alba* AHK119	A68V/S219P	p-nitrophenyl butyrate	7.0	37 °C	16 h	Activity: 50-fold increase	[55]
	TfCut2	*Thermobifida fusca*	D204C/E253C/D174R	Low crystallinity PET films	8.0	70 °C	48 h	Weight loss: 25.0 ± 0.8% (WT: 0.3 ± 0.1%)	[56]
	TfCut2	*Thermobifida fusca* KW3	G62A/I213S	Amorphous PET films	8.0	65 °C	50 h	Weight loss: 42% (2.7-fold)	[63]
	Cut190	*Saccharomonospora viridis* AHK190	Q138A/D250C-E296C/Q123H/N202H	Microfiber amorphous PET	8.5	70 °C	3 d	Degradation rate: > 30%	[70]
	PETase	*Ideonella sakaiensis* 201-F6	S121E/D186H/R280A, S121D/D186H, S121E/D186H	Commercial PET films	9.0	40 °C	24 h	Activity: 9.1-, 3.4-, and 4.5-fold, respectively	[71]
	PETase	*Ideonella sakaiensis* 201-F6	S121E/D186H/R280A, S121D/D186H, S121E/D186H	Commercial PET films	9.0	40 °C	72 h	Activity: 13.9-, 4.4-, and 6.0-fold, respectively	[71]
	PETase	*Ideonella sakaiensis* 201-F6	S242T, N246D, S121E/D186H/S242T/N246D	Bottle-grade PET films	9.0	37 °C	24 h	Activity: 2.5-, 2.6-, and 58-fold, respectively	[72]
	Cbotu_EstA	*Clostridium botulinum*	del71Cbotu_EstA	Amorphous PET films	7.0	50 °C	120 h	Activity: > 8-fold	[73]
Increasing the substrate accessibility	PETase	*Ideonella sakaiensis* 201-F6	S93M, W159F, N241F	L-naphthyl butyrate	8.0	30 °C		Activity: 2.5-, 4.3-, and 3.3-fold, respectively	[48]
	Thc_Cut1	*Thermobifida cellulosylitica*	Fusion to CBM	Amorphous PET films	7.0	50 °C	72 h	Productivity of TPA and MHET: 1.7 mol/mol (WT: 1.2 mol/mol)	[60]
	Thc_Cut1	*Thermobifida cellulosylitica*	Fusion to CBM	Amorphous PET films	7.0	50 °C	72 h	Productivity of TPA and MHET: 4.5 mol/mol (WT: 1.2 mol/mol)	[60]
	Thc_Cut1	*Thermobifida cellulosylitica*	Fusion to HFB4	Amorphous PET films	7.0	50 °C	24 h	Degradation rate: >16-fold	[61]
	Thc_Cut1	*Thermobifida cellulosylitica*	Fusion to HFB7	Amorphous PET films	7.0	50 °C	24 h	Degradation rate: >16-fold	[61]
	PETase	*Ideonella sakaiensis* 201-F6	R53E	Low crystallinity PET films	8.0, 9.0	30 °C		Degradation rate: >0.2 nmol/min^−1^cm^−2^	[65]
	TfCut2	*Thermobifida fusca* KW3	G62A/F209A	Low crystallinity PET films	9.0	65 °C	30 h	Degradation rate: 97 ± 1.8%	[74]
Reducing the interaction between enzymes and products	HiC	*Humicola insolens*		Non-carbonated mineral water bottles	7.0	50 °C	14 d	Degradation rate: 7.7-fold increase	[62]
	TfCut2	*Thermobifida fusca* KW3	G62A/I213S	Amorphous PET films	8.0	65 °C	50 h	Weight loss: 42% (2.7-fold)	[63]
	TfCut2	*Thermobifida fusca* KW3	TfCa-TfCut2	Amorphous PET films	8.0	60 °C	24 h	Productivity: increased by 91%	[75]
	LCC	Leaf-branch compost	TfCa-LCC	Amorphous PET films	8.0	60 °C	24 h	Total products: increased by 104%	[75]

^1^ All multiples without explanation are compared to wild-type (WT).

**Table 2:** Engineered microbial chassis for PET biodegradation.

Organism	Strain	Hydrolase	Strategy	Signal Peptide	Reaction Temperature	Substrate	Effects	Ref.
Bacteria	*B. subtilis* CBS2	BhrPETase	Overexpression of molecular chaperones (△ *hrcA*)	AprE	70 °C	Amorphous PET films	Expression titer: 0.66 g/L; 6.3 mM products (0.17 mM BHET, 3.66 mM MHET, 2.47 mM TPA) for 20 h	[42]
		LCC					Expression titer: 0.89 g/L	
	*E. coli* BL21(DE3)-T1R	PETase	Selection of signal peptides	SP_LamB_	30 °C	Commercial PET films	Expression titer: 6.2 mg/L; MHET/TPA: 2.3 mg/L for 24 h, 3.7 mg/L for 72 h	[80]
	*E. coli* BL21-Gold (DE3)	PETase	Selection and random mutagenesis of signal peptides	PM3 (Evolved PelB, G58A)	30 °C	BHET	Enzymatic activity: 1.7-fold more than PelB	[81]
						PET powder/	Enzymatic activity: produced 1117 μM MHET and TPA (2.1-fold more than PelB) for 18 h	
						Amorphous PET films	Enzymatic activity: obvious morphological changes and pores appeared for 168 h	
	*E. coli* BL21 (DE3)	PETase	Signal peptide modification	PelB modified by enhancer B1 (MERACVAV)	30 °C	Amorphous PET films	62-fold more excretion than PelB	[82]
			Fusion hydrophobin (HFBII)				MHET/TPA: 2.7-fold increase for 42 h	
	*B. subtilis* 168	PETase	Selection of signal peptides; Delete Tat translocases	SP_PETase_	30 °C	Amorphous PET films	3.8-fold more excretion than not deleted Tat translocases; Expression titer: 15 mg/L for 20 h;	[83]
	*B. subtilis* WB600	PETase	Selection of signal peptides; Promoter optimization (P43 promoter)	SP_amy_	28 °C	PET films	Pores and serious corrosion appeared for 36 h	[84]
	*C. thermocellum*	LCC	Thermophilic whole-cell degradation system	Signal peptide from exoglucanase Cel48S	60 °C	Commercial PET films	Soluble monomer feedstocks: >30 mg (60%) for 14 d	[85]
Fungi	*Y. lipolytica* Po1f	PETase	Selection of signal peptides	SP_LIP2_	30 °C	BHET	3.68 mM BHET was degraded for 1 h	[76]
	*P. pastoris*	PETase	Surface display; Whole-cell biocatalyst	ND	30 °C	Commercial PET bottles	Enzymatic activity: 36-fold increase	[86]
Marine microalgae	*P. tricornutum*	PETase^R280A^	Fusion proteins and localization	Alkaline phosphatase (AP)	21 °C	Commercial PET beverage bottle	Almost all MHET was converted into TPA for 10 d	[87]
	*C. reinhardtii* CC-124	PETase^R280A^	Cell lysis catalysis	ND	30 °C	Commercial PET beverage bottle	Many holes and dents appeared and TPA was detected for 4 weeks	[88]

## Data Availability

Not applicable.
